# Comparative Mitogenomic Analysis Reveals Intraspecific, Interspecific Variations and Genetic Diversity of Medical Fungus *Ganoderma*

**DOI:** 10.3390/jof8080781

**Published:** 2022-07-26

**Authors:** Qiang Li, Ting Zhang, Lijiao Li, Zhijie Bao, Wenying Tu, Peng Xiang, Qian Wu, Ping Li, Mei Cao, Wenli Huang

**Affiliations:** 1 Key Laboratory of Coarse Cereal Processing, Ministry of Agriculture and Rural Affairs, Sichuan Engineering & Technology Research Center of Coarse Cereal Industrialization, School of Food and Biological Engineering, Chengdu University, Chengdu 610106, China; liqiang02@cdu.edu.cn (Q.L.); z1377311342@126.com (T.Z.); j14736449833@126.com (L.L.); bzj13951294943@126.com (Z.B.); twy1712076841@126.com (W.T.); xiangpeng110@126.com (P.X.); w2394045772@126.com (Q.W.); 2 Biotechnology and Nuclear Technology Research Institute, Sichuan Academy of Agricultural Sciences, 106 # Shizishan Rd., Chengdu 610061, China; liping_0901@sina.com; 3 Core Laboratory, Sichuan Provincial People’s Hospital, University of Electronic Science and Technology of China, Chengdu 610072, China; 4 Chinese Academy of Sciences Sichuan Translational Medicine Research Hospital, Chengdu 610072, China

**Keywords:** mitochondrial genome, *Ganoderma*, gene rearrangement, intron, phylogenetic analysis, evolution

## Abstract

*Ganoderma* species are widely distributed in the world with high diversity. Some species are considered to be pathogenic fungi while others are used as traditional medicine in Asia. In this study, we sequenced and assembled four *Ganoderma* complete mitogenomes, including *G. subamboinense* s118, *G. lucidum* s37, *G. lingzhi* s62, and *G. lingzhi* s74. The sizes of the four mitogenomes ranged from 50,603 to 73,416 bp. All *Ganoderma* specimens had a full set of core protein-coding genes (PCGs), and the *rps3* gene of *Ganoderma* species was detected to be under positive or relaxed selection. We found that the non-conserved PCGs, which encode RNA polymerases, DNA polymerases, homing endonucleases, and unknown functional proteins, are dynamic within and between *Ganoderma* species. Introns were thought to be the main contributing factor in *Ganoderma* mitogenome size variation (*p* < 0.01). Frequent intron loss/gain events were detected within and between *Ganoderma* species. The mitogenome of *G. lucidum* s26 gained intron P637 in the *cox3* gene compared with the other two *G. lucidum* mitogenomes. In addition, some rare introns in *Ganoderma* were detected in distinct Basidiomycetes, indicating potential gene transfer events. Comparative mitogenomic analysis revealed that gene arrangements also varied within and between *Ganoderma* mitogenomes. Using maximum likelihood and Bayesian inference methods with a combined mitochondrial gene dataset, phylogenetic analyses generated identical, well-supported tree topologies for 71 *Agaricomycetes* species. This study reveals intraspecific and interspecific variations of the *Ganoderma* mitogenomes, which promotes the understanding of the origin, evolution, and genetic diversity of *Ganoderma* species.

## 1. Introduction

The genus *Ganoderma* belongs to the *Polyporaceae* family, *Polyporales* order, which is a group of fungi with high species diversity [1]. It is estimated that there are more than 180 species of the genus *Ganoderma* in the world [2,3,4,5]. Some species are identified as pathogenic fungi of woody plants, such as *G. boninense* [6], *G. steyaertanum* [7], *G. mastoporum* [8], and *G. philippii* [9], while some *Ganoderma* species are well-known components of traditional Asian medicine [10,11,12]. *G. lucidum*, *G. leucocontextum*, *G. sinense*, *G. lingzhi*, and other *Ganoderma* species have a variety of bioactive ingredients, which show great economic and medical value [13,14,15,16,17,18]. These bioactive ingredients of *Ganoderma* have attracted increasingly more attention in Western medicine and health industries, and its annual economic value reached several billion dollars in 2017 [2]. Due to the high similarity of basidiocarp features, the genus *Ganoderma* is the most difficult group to be accurately morphologically identified to species among all *Polyporales* [5,19,20]. The cultivation scale of *Ganoderma* species in Asia has continued to expand in recent years, and the phenomenon of confusion and misidentification of *Ganoderma* species is very prevalent, which limits the development and effective utilization of *Ganoderma* species [2,5,21]. The mitochondrial genome functions as a powerful tool in research of the phylogeny and evolution of fungal species [22,23]. However, so far, only nine complete mitochondrial genomes of *Ganoderma* species have been published [24,25,26,27], and the intraspecific and interspecific variation characteristics and genetic evolution of *Ganoderma* species have not been analyzed.

The mitochondrial genome is regarded as the ‘second genome’ of eukaryotes, which has an important function in eukaryote growth, development, and adaptation to the environment [28]. Animal mitochondrial genomes have been well studied because of their relatively conservative structure and gene content [29,30]. In comparison with animal mitochondrial genomes, fungal mitochondrial genomes vary greatly [31,32,33]. It is difficult to obtain complete mitochondrial genomes of fungi due to the genetic and structural variations [34,35]. Less than 130 complete mitochondrial genomes of Basidiomycetes have been published in the NCBI database (https://www.ncbi.nlm.nih.gov/genome/browse#!/organelles/ (accessed on 1 January 2022)), which is partly because mitochondrial data are ignored during genome assembly. With the rapid development of next-generation and third-generation high-throughput sequencing technologies, increasingly more complete fungal mitochondrial genomes will be obtained. The mitochondrial genomes of most fungal species were found to contain 14 core protein-coding genes, 20–36 tRNA genes, and 2 rRNA genes [36,37,38,39,40]. The gene arrangement, intron dynamic, repetitive sequences, expansion or contraction intergenic regions, and non-conserved mitochondrial genes provide a large amount of information that can be used to explore the origin and evolution of fungi [41,42,43,44]. We previously found that *Ganoderma* mitochondrial introns experienced loss or gain, and detected large-scale mitogenome rearrangements between different *Ganoderma* species [25]. Until now, the intraspecific variations of mitochondrial genomes in *Ganoderma* have not been fully revealed [45].

Herein, we performed sequencing and assembly of the mitochondrial genomes of four *Ganoderma* specimens, namely, *G. subamboinense* s118, *G. lucidum* s37, *G. lingzhi* s62, and *G. lingzhi* s74. We compared the four novel mitogenomes and published *Ganoderma* mitogenomes for the purpose of revealing the differences or similarities of *Ganoderma* mitogenomes in the size of the genome, content, arrangement of genes, and repeat sequences. Further, we determined the dynamic changes of introns in *Ganoderma* mitogenomes. We evaluated the phylogenetic relationships between *Ganoderma* species according to a combined mitochondrial gene set. The four novel *Ganoderma* mitogenomes promote our understanding of the intraspecific and interspecific variations, mitochondrial evolution, and genetic diversity of this important medical fungal genus.

## 2. Materials and Methods

### 2.1. Sequencing, Assembly, and Annotation of Mitogenomes

Four *Ganoderma* specimens were collected from Sichuan Province in southwest China, which were then stored in Culture Collection Center of Sichuan Academy of Agricultural Sciences (Contact information: Wenli Huang, wenlih11@126.com), including *G. subamboinense* s118, *G. lucidum* s37, *G. lingzhi* s62, and *G. lingzhi* s74. The growth environment information and collection information of these specimens can be found in Supplementary Table S1. We extracted the genomic DNA of the four *Ganoderma* specimens using a fungal DNA extraction kit (Omega Bio-Tek, Norcross, GA, USA). Sequencing libraries were constructed with the genomic DNA using a NEBNext^®^ Ultra™ II DNA Library Prep Kit (NEB, Beijing, China) according to the instructions. We conducted whole genomic sequencing (WGS) using the Illumina HiSeq 2500 Platform (Illumina, San Diego, CA, USA). In order to process the raw data, a series of quality control steps were carried out. Among these steps, ngsShoRT was used for filtering low-quality sequences [46] and AdapterRemoval v2 was used to remove adapter reads [47]. We *de novo* assembled the four *Ganoderma* mitogenomes using NOVOPlasty v3.7.2 [48] with a k-mer size of 25. The four mitogenomes obtained were annotated using the MFannot tool [49] and MITOS [50] according to our previously described methods [51]. We drew the circular maps of the four *Ganoderma* mitogenomes using OGDRAW [52].

### 2.2. Sequence Analysis

The base compositions of the four *Ganoderma* mitogenomes were analyzed using DNASTAR Lasergene v7.1 (http://www.dnastar.com/ (accessed on 1 January 2022)). We determined the four mitogenomes’ strand asymmetry using the formulas as follows: AT skew = [A − T]/[A + T] and GC skew = [G − C]/[G + C] [53]. We determined the genetic distances between the pairs among the 15 core protein-coding genes (PCGs)—*atp6*, *atp8*, *atp9*, *cob*, *cox1*, *cox2*, *cox3*, *nad1*, *nad2*, *nad3*, *nad4*, *nad4L*, *nad5*, *nad6*, and *rps3*—using MEGA v6.06 [54] following the Kimura-2-parameter (K2P) model. We used DnaSP v6 to compute the nonsynonymous substitution rate (*Ka*) and the synonymous substitution rate (*Ks*) for the 15 core PCGs [55]. The Sequence Manipulation Suite [56] following the genetic code 4 conducted the codon usage analysis. BLASTn searching of the four mitogenomes against themselves at an E value of <10^−10^ was carried out in order to establish whether there were intra-genomic duplications or interspersed repeats (>30 bp) in the four *Ganoderma* mitogenomes. In addition, tandem repeats (>10 bp) in the four mitogenomes were identified with Tandem Repeats Finder [57].

### 2.3. Intron Analysis

Fungal mitochondrial introns vary greatly, and usually do not contain any introns in most eukaryotic mitogenomes while basidiomycetes, including *Polyporales*, usually contain varying numbers of introns [58,59]. We further separated the 15 core PCG introns in *Ganoderma* and other *Polyporales* mitogenomes (the accession numbers are shown in Table S7) into position classes (Pcls) with the mitogenome of *Ganoderma calidophilum* [25] as a reference in accordance with reported methods [60]. First, the core PCGs excluding introns were aligned with reference mitochondrial sequences of *G. calidophilum* using Clustal W [61]. Each Pcl comprised introns inserted at the same position in the PCG coding regions [60]. Introns that are of the same Pcl typically have high sequence similarities and are deemed orthologous [62]. Most Pcls that differ possess low sequence similarities and non-orthologous mobile genetic elements [45]. Pcls of 15 core PCGs in *Polyporales* were given names based on the reference host gene’s coding region insert position.

### 2.4. Phylogenetic Analysis

A phylogenetic tree of 71 *Agaricomycetes* species was constructed based on the combined mitochondrial gene set in order to explore the phylogenetic relationships of *Ganoderma* species and the phylogenetic status of these *Ganoderma* species among the *Agaricomycetes* class (the accession numbers are shown in Table S7). The mitogenome of *Hannaella oryzae* from the order *Tremellales* was designated as an outgroup [63]. The combined mitochondrial gene set contains 14 core protein-coding gene sequences, excluding the *rps3* gene and intron regions. For alignment of single mitochondrial genes, we used MAFFT v7.037 [64]. We subsequently used SequenceMatrix v1.7.8 to concatenate the resulting alignments into a gene set [65]. We employed PartitionFinder 2.1.1 to identify the gene set’s best-fit evolution and partitioning scheme models [66]. MrBayes v3.2.6 was used [67] along with the Bayesian inference (BI) method based on the combined gene set to evaluate the phylogenetic relationships between *Agaricomycetes* species. BI analysis involved two independent runs with three heated chains and one cold chain, each performed simultaneously for 2 × 10^6^ generations. Once every 1000 generations, each run was sampled. Stationarity was assumed to have been achieved when the estimated sample size (ESS) reached more than 100, and the potential scale reduction factor (PSRF) became close to 1.0 (the closer the PSRF value was to 1, the better the convergence effect). We eliminated the first 25% of samples as burn-in. Then, we made use of the remaining trees to evaluate the Bayesian posterior probabilities (BPPs) in a 50% majority-rule consensus tree [68]. In addition, the maximum likelihood (ML) technique was employed for assessment of the phylogenetic relationships of 71 *Agaricomycetes* species using RAxML v8.0.0 [69] with the combined gene set. We performed an evaluation of the bootstrap values (BS) using an ultrafast bootstrap approach with 1000 replicates.

## 3. Results

### 3.1. Characterization of the Four Ganoderma Mitogenomes

**Figure 1:**
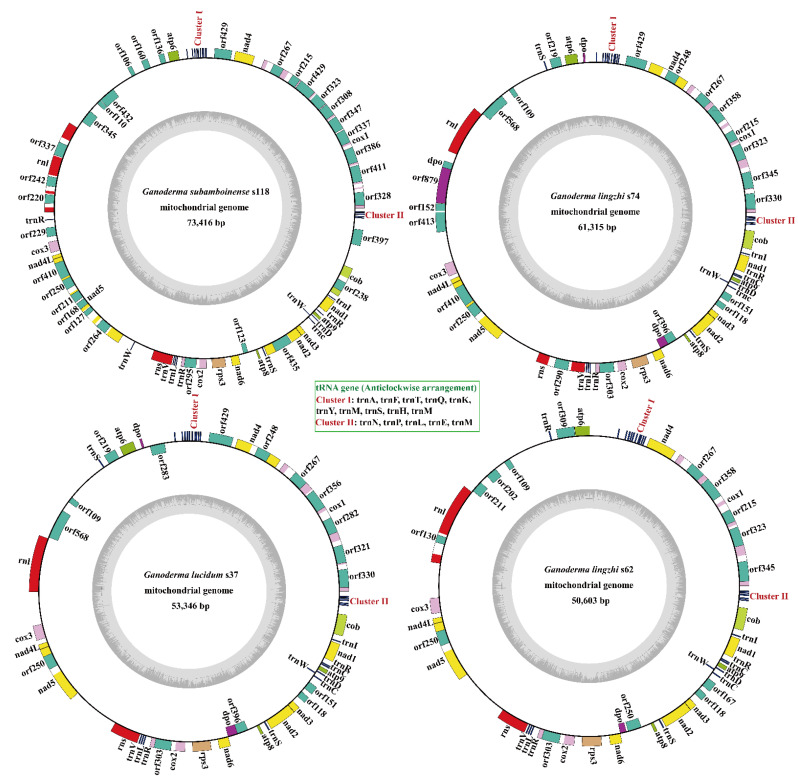
Circular maps of the four *Ganoderma* mitogenomes. Genes are represented by different colored blocks. Colored blocks outside each ring indicate that the genes are on the direct strand while colored blocks within the ring indicate that the genes are located on the reverse strand.

The four complete *Ganoderma* mitogenomes were all made up of circular DNA molecules that ranged in size from 50,603 to 73,416 bp (Figure 1), and the molecular structure was further verified by polymerase chain reaction amplification and pyrophosphate sequencing. The mitogenome of *G. subamboinense* s118 was the largest while the *G. lingzhi* s74 was the smallest among the four *Ganoderma* mitogenomes. The GC content of the four mitogenomes was within 26.33–26.56%, with a 26.41% average GC content (Table S1). The mitogenomes of *G. subamboinense* s118 and *G. lucidum* s37 exhibited positive AT skews while the two strains of *G. lingzhi* had negative AT skews. The GC skews of the four *Ganoderma* mitogenomes were all positive. In total, 23 to 28 non-intronic open-reading frames (ORFs) were detected in the 4 *Ganoderma* mitogenomes, which included 15 core PCGs and 8–12 free-standing ORFs. These free-standing ORFs mainly encoded DNA polymerases, RNA polymerases, and proteins with unknown functions in the four *Ganoderma* mitogenomes (Table S2). A total of 48 introns were detected in the 4 *Ganoderma* mitogenomes, with each containing 8–21 introns. Each intron contained one or two intronic ORFs, encoding *LAGLIDADG* homing endonucleases or *GIY-YIG* homing endonucleases. A total of 49 intronic ORFs were detected in the 4 *Ganoderma* mitogenomes. Among the 48 introns detected in the 4 *Ganoderma* specimens, 29 belonged to Group IB, 7 belonged to Group IA, 6 belonged to Group ID, 4 belonged to Group IC2, 1 belonged to Group II, and 1 was an unknown type. Two rRNA genes were found in all four *Ganoderma* mitogenomes, namely, the small subunit ribosomal RNA (*rns*) and the large subunit ribosomal RNA (*rnl*). In addition, the 4 *Ganoderma* mitogenomes all contained 26 tRNA genes.

### 3.2. Codon Usage Analysis

Up to now, nine complete mitochondrial genomes of *Ganoderma* species have been published in the database (the accession numbers are shown in Table S7). We further compared these 13 *Ganoderma* mitogenomes to reveal the conservation and variation between *Ganoderma* mitogenomes. Of the 15 core PCGs in the 13 *Ganoderma* mitogenomes we detected, 14 used ATG as start codons except for *cob*, which used GTG as start codons in all the 13 *Ganoderma* species (Table S3). The majority of the core PCGs in thee *Ganoderma* species used TAA as stop codons while the *atp6* gene of *G. calidophilum*, *G. applanatum*, and *G. subamboinense* s118, and the *cox2* gene of *G. calidophilum* used TAG as stop codons. The *nad1* and *nad2* genes of most *Ganoderma* species used TAG as stop codons.

**Figure 2:**
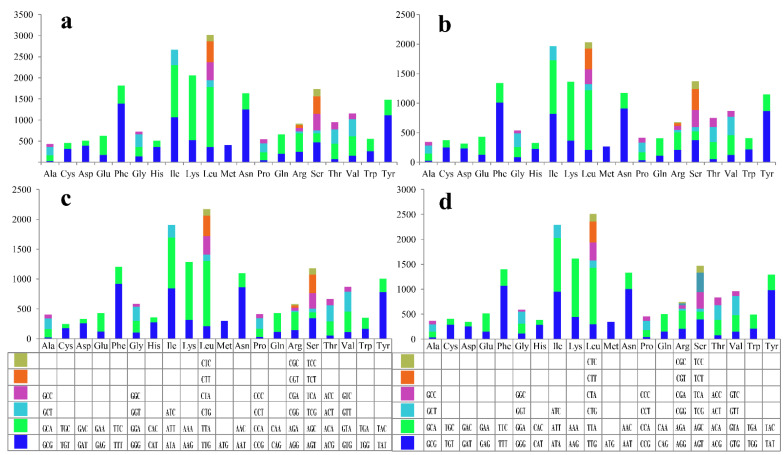
Codon usage analysis of the four newly sequenced *Ganoderma* mitogenomes: (**a**) *G. subamboinense* s118; (**b**) *G. lucidum* s37; (**c**) *G. lingzhi* s62; and (**d**) *G. lingzhi* s74.

Codon usage analysis revealed that the most frequently used codons were AAA in the *G. subamboinense* s118 and *G. lingzhi* s74 mitogenomes, TTT in *G. lucidum* s37, and TTA in the *G. lingzhi* s62 mitogenome (Figure 2 and Table S4). In general, AAA (for lysine; Lys), TTA (for leucine; Leu), TTT (for phenylalanine; Phe), AAT (for asparagine; Asn), and ATT (for isoleucine; Ile) were the five most frequently used codons in the four newly sequenced *Ganoderma* mitogenomes. The high AT content in the four *Ganoderma* mitogenomes (average: 73.59%) was due to frequently used codons ending in A and T.

### 3.3. Repetitive Sequences Analysis

We identified 47, 9, 7, and 7 repeat sequences in the mitogenomes of *G. subamboinense* s118, *G. lucidum* s37, *G. lingzhi* s62, and *G. lingzhi* s74, respectively, by comparison of the whole mitogenomes against themselves via BLASTn analysis (Table S5). In the four *Ganoderma* mitogenomes, repeat sequence lengths ranged from 35 to 868 bp while the range of pair-wise nucleotide similarities was 66.07–100%. The *orf410*, *orf211* and *orf168* protein-coding regions had the largest repeats, and in the intergenic region between *orf211* and *orf168* in the *G. subamboinense* mitogenome. The repetitive sequences comprised 2.33–12.12% of the whole mitogenomes of the four *Ganoderma* specimens. The *G. subamboinense* s118 mitogenome exhibited the highest proportion of repeat sequences while the lowest proportion of repeat sequences was found in the *G. lingzhi* s74 mitogenome.

A total of ten, four, two, and four tandem repeats were found in the mitogenomes of *G. subamboinense* s118, *G. lingzhi* s74, *G. lucidum* s37, and *G. lingzhi* s62, respectively (Table S6). The mitogenome of *G. subamboinense* s118 contained the longest tandem repeat sequence at 45 bp. Among the four newly sequenced mitogenomes, most of the tandem repeats were duplicated once or twice, with the highest replication number (12) in the mitogenome of *G. subamboinense* s118. Tandem repeat sequences made up 0.14–0.49% of the four *Ganoderma* mitogenomes.

### 3.4. Variation, Genetic Distance, and Evolutionary Rates of Core Genes

**Figure 3:**
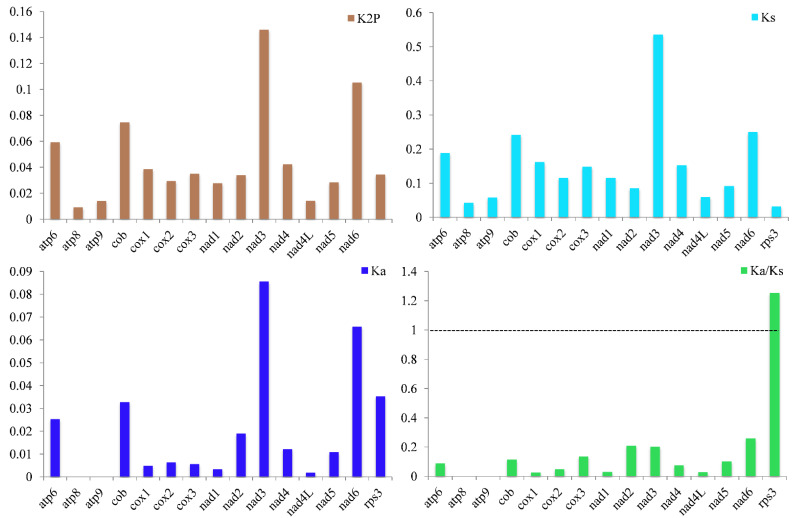
Genetic analysis of 15 protein-coding genes conserved in the 13 *Ganoderma* mitogenomes. K2P, the Kimura-2-parameter distance; Ka, the mean number of nonsynonymous substitutions per nonsynonymous site; Ks, the mean number of synonymous substitutions per synonymous site.

Of the 15 core PCGs revealed herein, the *nad3* gene and then the *nad6* gene showed the greatest mean K2P genetic distance between the 13 *Ganoderma* species (Figure 3). On average, the *atp8* gene exhibited the smallest K2P genetic distance between the 13 *Ganoderma* species, showing the *atp8* gene was highly conserved. The *nad3* gene exhibited the greatest mean nonsynonymous substitution rate (*Ka*) while the *atp8* and *atp9* genes had the lowest *Ka* values. The *nad3* gene revealed the highest synonymous substitution rate (*Ks*) among the 15 core PCGs while that of the *rps3* gene was the lowest. The *Ka/Ks* values of 14 out of 15 core PCGs were less than 1, demonstrating that these genes underwent purifying selection. However, the *Ka/Ks* values of the *rps3* gene had values greater than one between some species, such as between *G. calidophilum* and *G. lucidum* s26, between *G. subamboinense* s118 and *G. leucocontextum*, and between *G. meredithae* and *G. sinense*, indicating that in some *Ganoderma* species, the *rps3* gene was under either positive or relaxed selection.

### 3.5. Intron Dynamic Analysis

**Figure 4:**
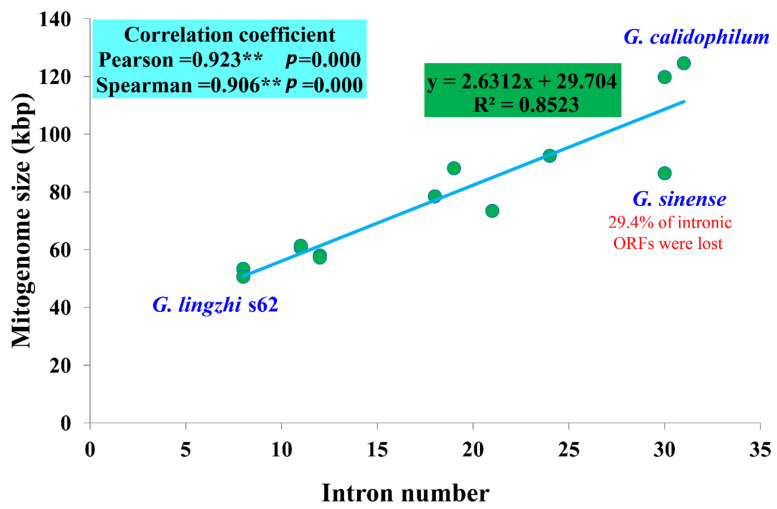
Correlation analysis between the number of intron and mitogenome sizes of 13 *Ganoderma* species. The species and NCBI accession numbers for the mitogenomes used in the correlation analysis are provided in Supplementary Table S7.

A significant correlation was found between the number of introns and the mitogenome sizes of 13 *Ganoderma* species (*p* < 0.01). The Pearson and Spearman correlation coefficients were 0.923 and 0.906, respectively, indicating that introns are the main factor causing the size variations of *Ganoderma* mitogenomes (Figure 4). A total of 235 introns were detected in the 13 *Ganoderma* mitogenomes we tested, with each containing 8–31 introns. The dynamic changes in the introns of *Ganoderma* species indicated that the loss or gain of introns occurred during the evolution of *Ganoderma* species. In total, 215 out of the 235 introns (91.49%) were distributed in core PCGs of the 13 *Ganoderma* species, indicating that the core PCGs were the largest host genes of introns in *Ganoderma*. The remaining 20 introns were distributed in rRNA genes. The introns of core PCGs were harbored in thee *cox1*, *cox2*, *cox3*, *cob*, *nad1*, *nad2*, *nad3*, *nad4*, and *nad5* genes, and the *cox1* gene contained the largest number of introns, with 114. Six core PCGs, including the *atp6*, *atp8*, *atp9*, *nad4L*, *nad6*, and *rps3* genes, did not contain any intron in the thirteen *Ganoderma* mitogenomes. The uneven distribution of introns in different core PCGs indicated that fungal introns had host gene preference. Since most of *Ganoderma* introns are distributed in the core PCGs, we studied the dynamic changes in the introns of *Ganoderma* core PCGs.

**Figure 5:**
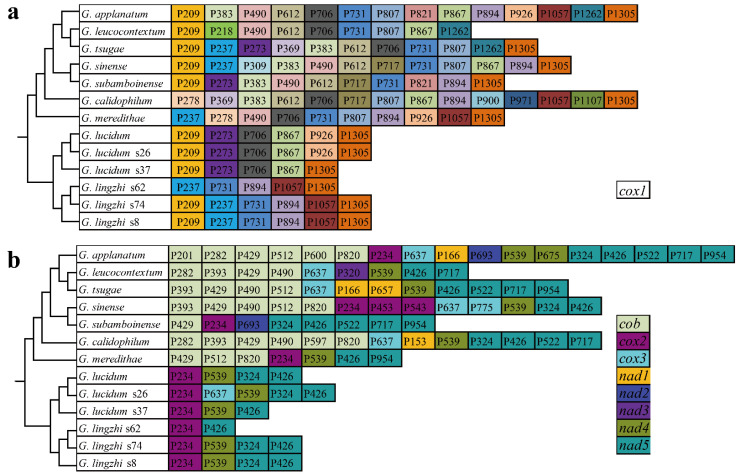
Position class (Pcl) information of *cox1* (**a**) and other core PCGs (**b**) of the 13 *Ganoderma* species. Pcls (orthologous introns) were named according to the insert sites (nt) in aligned reference genes (MH252535). The phylogenetic positions of the 13 *Ganoderma* species were established using the Bayesian inference (BI) method and maximum likelihood (ML) method based on a combined mitochondrial gene set.

Using the insertion sites of introns in the protein-coding region of host genes as a basis, introns were categorized into different position classes (Pcls) depending on their corresponding reference genes. The 114 introns of the *cox1* gene could be divided into 24 Pcls in the 13 *Ganoderma* mitogenomes (Figure 5). P1305 was the Pcl that was most broadly distributed; it was distributed in 12 of the 13 *Ganoderma* species. P209 was also widely distributed in *Ganoderma cox1* genes, which was distributed in 10 of the 13 *Ganoderma* species. Some rare intron was only distributed in 1 of the 13 *Ganoderma cox1* genes, including P218, P309, P900, P971, and P1107. We also detected nine, three, two, three, one, one, two, and five Pcls in the *cob*, *cox2*, *cox3*, *nad1*, *nad2*, *nad3*, *nad4*, and *nad5* genes. P426 from the *nad5* gene was the most common intron in *Ganoderma* species, which was present in all of the 13 *Ganoderma* mitogenomes. P234 from the *cox2* gene was also widely distributed in *Ganoderma* species, which was distributed in 10 of the 13 *Ganoderma* mitogenomes. However, P201, P597, and P600 from the *cob* gene; P453 and P543 from the *cox2* gene; P775 from the *cox3* gene; P153 and P657 from the *nad1* gene; P320 from the *nad3* gene; and P675 from the *nad4* gene were only detected in 1 of the 13 *Ganoderma* species, which were considered to be rare Pcls in *Ganoderma.* We found that these rare Pcls were also distributed in distant species, such as *Paxillus rubicundulus* [51], *Agaricus bisporus* [62], *Rhizopogon salebrosus* [31], *Russula compacta* [37], and *Moniliophthora perniciosa* [70], suggesting that potential intron transfers may take place in *Ganoderma* mitogenomes, or that intron insertions were convergent in distantly related species.

We observed loss or gain of introns within *G. lucidum* and *G. lingzhi* species. The *G. lucidum* s37 lost P926 in the *cox1* gene, and P324 in the *nad5* gene compared with the other two *G. lucidum* mitogenomes. The mitogenome of *G. lucidum* s26 gained P637 in the *cox3* gene compared with the other two *G. lucidum* mitogenomes. Within the *G. lingzhi* species, the *G. lingzhi* s62 lost P209 in the *cox1* gene, P539 in the *nad4* gene, and P324 in the *nad5* gene compared with the other two *G. lingzhi* mitogenomes.

### 3.6. Comparative Mitogenomic Analysis

Comparative genomic analysis showed that the mitogenome of *G. calidophilum* was the largest among the 13 *Ganoderma* mitogenomes while *G. lingzhi* and *G. lucidum* had the smallest mitogenome among the 13 *Ganoderma* species, with an average size of 56,383 and 57,304, respectively (Table S1). The mitogenome size also varied within the *G. lingzhi* and *G. lucidum* species. The GC content was also found to vary between and within *Ganoderma* species, indicating base composition dynamics in the evolution of *Ganoderma* species. Base biases were frequently detected in the mitogenomes of *Ganoderma*. The AT and GC skews of *G. leucocontextum* and AT skew of *G. lucidum* (KC763799) were negative (T over A; C over G) in their mitogenomes. However, there was an excess of Gs (but not Cs) and As (but not Ts) in the replication leading strands of most *Ganoderma* mitogenomes. Different *Ganoderma* species contained varied numbers of non-conserved ORFs, encoding DNA polymerase, RNA polymerase, homing endonucleases, and unknown functional proteins (Table 1). The six *G. lucidum* and *G. lingzhi* strains all contained the *orf109,* which encoded RNA polymerase. The *orf283* encoding DNA polymerase could only be detected in *G. lucidum*. Some non-conserved PCGs only existed in the mitogenome of *G. lingzhi*, including *orf115*, *orf250*, *orf744*, and *orf879* encoding DNA polymerases; *orf202* and *orf211* encoding RNA polymerases; *orf118* and *orf309* encoding homing endonucleases; and *orf152* and *orf413* encoding unknown functional proteins. In *G. lucidum*, strain s37 lost *orf151*, *orf192*, and *orf294*, which encoded unknown functional proteins, while strain s26 lost *orf283*, which encoded DNA polymerase. In *G. lingzhi*, the *orf118* encoding *GIY-YIG* endonuclease was lost in strain s8 while the strain s26 gained the *orf115* and *orf250* encoding DNA polymerases, and *orf202* and *orf211* encoding RNA polymerases compared with other *G. lingzhi* species. These results indicate that loss or gain events of non-conserved PCGs have occurred in the evolution of *Ganoderma*. The position classes and number of introns also varied within and between *Ganoderma* species. In addition, 29.41% of the intronic ORFs in the *G. sinense* mitogenome have been lost, indicating that intron ORFs of *G. sinense* are undergoing contraction. The number of tRNA in *Ganoderma* mitogenomes varied between *Ganoderma* species but was conserved within *Ganoderma* species. All *Ganoderma* species contained two mitochondrial rRNA genes.

### 3.7. Gene Arrangement and Phylogenetic Analyses

**Figure 6:**
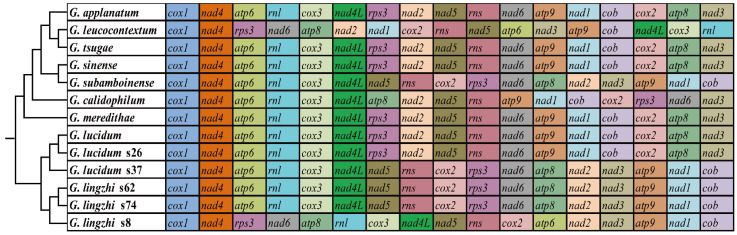
Gene order comparation between 13 *Ganoderma* species. All genes are shown in order of occurrence in the mitochondrial genome, starting from *cox1*. Fifteen core protein-coding genes and two rRNA genes were included in the gene arrangement analysis. The phylogenetic positions of 13 *Ganoderma* species were established using the Bayesian inference (BI) method and maximum likelihood (ML) method based on concatenated mitochondrial genes. Species and NCBI accession number used for gene arrangement analysis in the present study are listed in Supplementary Table S7.

The organization of 15 core PCGs and 2 rRNA genes in the 13 *Ganoderma* mitogenomes was compared. We found that the gene order of 13 *Ganoderma* species varied greatly between species, even within species (Figure 6). The mitogenomes of *G. applanatum*, *G. tsugae*, *G. sinense*, *G. meredithae*, *G. lucidum* (KC763799), and *G. lucidum* s26 had identical gene arrangements, which were arranged in the following order: *cox1*, *nad4*, *atp6*, *rnl*, *cox3*, *nad4L*, *rps3*, *nad2*, *nad5*, *rns*, *nad6*, *atp9*, *nad1*, *cob*, *cox2*, *atp8*, and *nad3*, while the mitogenomes of *G. subamboinense* s118, *G. lucidum* s37, *G. lingzhi* s62, and *G. lingzhi* s74 had another gene order: *cox1*, *nad4*, *atp6*, *rnl*, *cox3*, *nad4L*, *nad5*, *rns*, *cox2*, *rps3*, *nad6*, *atp8*, *nad2*, *nad3*, *atp9*, *nad1*, and *cob*, which were different from other *Ganoderma* species. Gene rearrangements within *G. lucidum* and *G. lingzhi* were observed, including gene transfer, insertion, and inversion events. The findings showed that large-scale gene rearrangements took place during the evolution of *Ganoderma* species.

**Figure 7:**
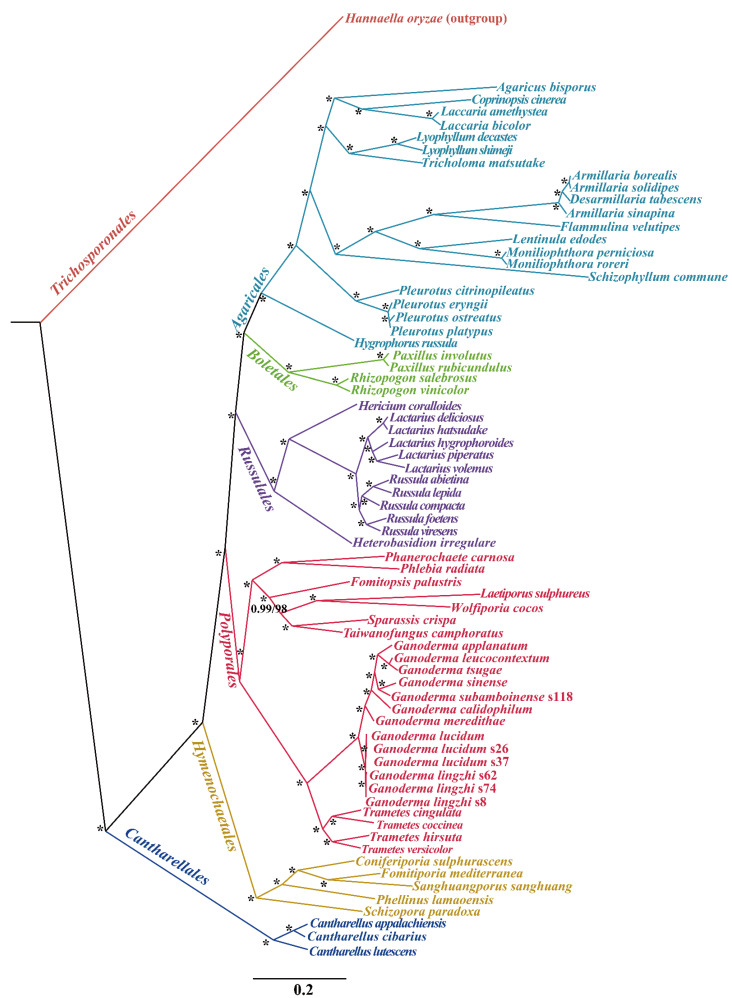
Phylogeny of 71 *Agaricomycetes* species based on 14 protein-coding genes using the Bayesian inference (BI) and maximum likelihood (ML) methods. The phylogenetic trees were generated by MrBayes v3.2.6 and then exported by FigTree v1.4.3. Support values are Bayesian posterior probabilities (before slash) and bootstrap (BS) values (after slash). The asterisk indicates that the BPP value is 1 and the BS value is 100 of the clades. The species and NCBI accession numbers for the mitogenomes used in the phylogenetic analysis are provided in Supplementary Table S7.

We performed phylogenetic analysis using maximum likelihood (ML) and Bayesian inference (BI) according to the combined mitochondrial gene set (14 core PCGs). These methods generated identical, well-supported tree topologies (Figure 7). All major clades within the phylogenetic tree were well supported (BPP ≥ 0.99; BS ≥ 98). According to the phylogenetic tree, the 71 *Agaricomycetes* species comprised 6 major clades of the orders *Agaricales*, *Boletales, Russulales*, *Polyporales*, *Hymenochaetales*, and *Cantharellales* (Table S7). The 24 *Polyporales* species could be divided into 7 groups, corresponding to the families *Phanerochaetaceae*, *Meruliaceae*, *Fomitopsidaceae*, *Laetiporaceae*, *Sparassidaceae*, and *Polyporaceae* while *Taiwanofungus* could not be placed in any recognized family. *Ganoderma* and *Tremetes* are closely related and form a group *Polyporaceae*. The phylogenetic analyses showed that *G. lingzhi* was a sister species to *G. lucidum*.

## 4. Discussion

### 4.1. Mitogenome Size Variation in Ganoderma

In the present study, the mitogenome size of *Ganoderma* species was found to vary greatly, ranging from 50,603 to 124,588 bp. The largest mitogenome *G. calidophilum* [25] in the genus *Ganoderma* is 2.46 times larger than the smallest one *G. lingzhi* s62. Previous studies have found that the fungal mitogenome sizes is one of the most variable groups in eukaryotes, which is mainly due to the dynamic changes in introns, the accumulation of repeat sequences, the content of non-conserved PCGs, and so on [71,72,73,74]. The number of introns in this study had a significant effect on the size variation of *Ganoderma* mitogenomes (*p* < 0.01). This suggests that introns may be the main contributing factor affecting the size variation of *Ganoderma* species. The mitogenomes of *G. lucidum* and *G. lingzhi* were the smallest among *Ganoderma* species, which indicated that the two species may have experienced intron loss events during evolution. We observed size variations of the mitogenomes within *G. lucidum* (7289 bp variations) and *G. lingzhi* (10,712 bp variations) species. Within the *G. lucidum* species, *G. lucidum* (KC763799) contained the largest mitogenome. However, the mitogenome of *G. lucidum* (KC763799) did not have the largest number of introns or non-conserved PCGs. Through comparative mitogenomic analysis, we found that the expansion or contraction of the intergenic region may explain the size variations within *G. lucidum* mitogenomes. In *G. lingzhi* species, the expansion of non-conserved PCGs can explain the size increase of the *G. lingzhi* s74 mitogenome. In general, the size variations of the *Ganoderma* mitogenomes are caused by several factors, including the dynamic changes in the introns, the expansion or contraction of the intergenic region, and the accumulation of non-conserved PCGs.

### 4.2. Mitogenome Content Evolution in Ganoderma

Since the ancestors of eukaryotes acquired mitogenomes from *Alphaproteobacteria* through endosymbiosis, most mitochondrial genes have been integrated into the nuclear genome during evolution [75,76]. However, most fungal mitogenomes retain a set of core PCGs and a number of non-conserved PCGs to ensure the stability of the oxidative phosphorylation process. Partial deletion of core PCGs was also observed in some fungal mitogenomes, indicating the complex evolution of fungal mitogenomes [45]. In this study, we found that all the mitogenomes of *Ganoderma* contained a whole set of core PCGs. The length, base composition, codon usage frequency, and start and stop codons of these core PCGs varied greatly within and between *Ganoderma* species, indicating that the core PCGs of *Ganoderma* mutated frequently. Interestingly, we found that the *rps3* gene demonstrated positive selection or relaxed selection between some *Ganoderma* species. This phenomenon has also been observed in other fungal species [77,78], and what causes the selection pressure on the *rps3* gene needs to be further verified. Different numbers of non-conserved PCGs were observed in the mitogenomes of *Ganoderma*, which encoded RNA polymerase, DNA polymerase, homing endonuclease, and unknown functional proteins. Two closely related *Ganoderma* species, *G. lucidum* and *G. lingzhi*, both contained *orf109* encoding RNA polymerase, which indicated that *orf109* may play an important role in the mitochondrial function of the two *Ganoderma* species. Interspecific differences in the non-conserved PCGs were detected between the two closely related *Ganoderma* species. All the three mitogenomes of *G. lucidum* contained *orf396*, *orf429*, and *orf568*, which encoded DNA polymerase, unknown functional protein, and RNA polymerase, respectively. However, *G. lingzhi* s26 lost the three non-conserved PCGs. All *G. lingzhi* contained an *orf151* gene, which encoded unknown functional protein, while *G. lucidum* s37 lost this gene. The dynamic changes in the RNA polymerase, DNA polymerase, homing endonuclease, and unknown functional genes were frequently observed within *G. lucidum* and *G. lingzhi* species, indicating that the loss and gain of non-conserved PCGs occurred in the evolution of *Ganoderma* mitogenomes, even within species.

### 4.3. Dynamics of Introns in Ganoderma

The dynamic change in the introns is thought to be the primary factor causing the size variation of the *Ganoderma* mitogenomes. Compared with mitogenomes from other genera, most of the intronic ORFs in *Ganoderma* species were retained, and only *G. sinense* lost 29.41% of the intronic ORFs [25]. It was found that introns were unevenly distributed in the *Ganoderma* core PCGs and rRNA genes, and the *cox1* gene was the largest host gene of introns in *Ganoderma*. Introns can be categorized into different Pcls depending on their insertion sites, and the same Pcls were considered to be homologous [79]. There was no significant correlation between the number of Pcls and the length of host genes, indicating that intron insertion sites had sequence selection preference. P209 and P1305 of the *cox1* gene, P234 of the *cox2* gene, and P426 of the *nad5* gene were considered to be the most common Pcls in *Ganoderma*, which were distributed in more than 10 of the 13 *Ganoderma* species. However, some rare Pcls were only detected in 1 of the 13 *Ganoderma* species, including P201, P597, and P600 from the *cob* gene; P453 and P543 from the *cox2* gene; P775 from the *cox3* gene; P153 and P657 from the *nad1* gene; P320 from the *nad3* gene; and P675 from the *nad4* gene. These rare Pcls were found in distant species, which indicated possible horizontal gene transfer events. In previous studies, introns were found to be horizontally transferred between different organelles [80]. We also detected intron loss and gain events in *G. lucidum* and *G. lingzhi*. The results show that the introns of *Ganoderma* are highly variable within and between species, and the physiological and functional effects of these intron dynamics need to be further revealed.

### 4.4. Gene Rearrangements and Phylogenetic Analysis

This research work found that the *Ganoderma* mitochondrial gene arrangement varied greatly within and between species. Some species with distant phylogenetic relationships had identical gene arrangements while some closely related species had varied gene arrangements, suggesting that the mitochondrial gene arrangement in *Ganoderma* species is both random and dynamic. Previous studies found that gene rearrangements occur frequently in fungal mitogenomes [81]. However, the fungal gene rearrangement mechanism remains unknown. Animal mitochondrial gene rearrangement has been investigated in depth, and researchers have designed multiple models to explore the mechanism of animal mitochondrial gene rearrangement [82,83]. The accumulation of repetitive sequences in fungal mitogenomes was believed to cause gene rearrangements in fungal mitogenomes [84]. However, the result cannot explain the intraspecific gene rearrangements of *Ganoderma* mitogenomes. More intraspecific mitochondrial gene rearrangements and their mechanisms need to be elucidated to understand the evolutionary patterns of fungal mitogenomes.

The *Ganoderma* species is widely distributed on Earth, with high species diversity [1,85]. Some *Ganoderma* species are pathogenic fungi of trees while some are traditional Asian medicinal materials [2,86]. The discovery of bioactive natural products from *Ganoderma* has attracted the enthusiasm of medical researchers all over the world and promoted the continuous expansion of the *Ganoderma* cultivation scale [87,88]. However, the limited and overlapped morphological features led to confusion in the accurate classification of *Ganoderma* species [5,19]. The phenomenon of synonyms and homonyms is very prevalent in cultivation and product development of *Ganoderma* species, which limits the large-scale development and utilization of this important medicinal fungus [20,21]. The mitogenome is an effective tool to understand the phylogenetic relationship of species [89,90,91]. However, the lack of a mitochondrial reference genome of *Agaricomycetes*, especially *Ganoderma* species, limits the application of the mitogenome in the classification and phylogenetic relationship analysis of *Ganoderma*. In this study, we newly obtained four complete mitogenomes of *Ganoderma* species. Based on the ML and BI phylogenetic inference methods, we obtained phylogenetic trees for 71 *Agaricomycetes* species with high support rates in major clades. The phylogenetic relationships among the *Ganoderma* species and the phylogenetic status of *Ganoderma* in *Agaricomycetes* were also revealed. The phylogenetic tree of the 24 *Polyporales* species based on the mitogenome is highly consistent with that based on polygenic molecular markers [92,93,94]. This study provides useful reference data for the classification and identification of *Ganoderma* species and promotes our understanding of intraspecific and interspecific variations of *Ganoderma* species.

## 5. Conclusions

In the present study, the mitogenomes of four *Ganoderma* specimens, including *G. subamboinense* s118, *G. lucidum* s37, *G. lingzhi* s62, and *G. lingzhi* s74, were sequenced and assembled. We performed comparative mitogenomic analysis of intraspecific and interspecific *Ganoderma* species to reveal the conservation and variation of *Ganoderma* mitogenomes. The sizes of *Ganoderma* mitogenomes varied within and between *Ganoderma* species, and the intron was the main factor contributing to the size variations of *Ganoderma* mitogenomes. Frequent intron loss/gain events were detected within and between *Ganoderma* species. The mitogenome of *G. lucidum* s26 gain intron P637 in *cox3* gene compared with the other two *G. lucidum* mitogenomes. The core PCG *rps3* of the *Ganoderma* species experienced positive or relaxed selection while non-conserved PCGs are dynamic within and between *Ganoderma* species. Large-scale gene arrangements were detected even within *Ganoderma* species. The results showed that there was high mitogenome diversity within and between *Ganoderma* species, regarding intron types, non-conserved genes, and gene arrangement. This study revealed intraspecific and interspecific variations of *Ganoderma* mitogenomes for the first time, which promotes the understanding of the evolution and genetic diversity of *Ganoderma* species.

## Figures and Tables

**Table 1:** Non-conserved protein-coding genes in the mitogenomes of *Ganoderma lucidum* and *G. lingzhi*.

PCGs	*G. lucidum*			*G. lingzhi*			Function
	GL	s26	s37	s62	s74	s8	
***orf109***	*	*	*	*	*	*	RNA polymerase
***orf115***	/	/	/	*	/	/	DNA polymerase
***orf118***	/	/	/	*	*	/	GIY-YIG endonuclease
***orf151***	*	*		*(*orf167*)	*	*	hypothetical protein
***orf152***	/	/	/	/	*	/	hypothetical protein
***orf190***	/	/	*	/	*	/	DNA polymerase
***orf192***	*	*	/	/	/	*	hypothetical protein
***orf202***	/	/	/	*	/	/	RNA polymerase
***orf211***	/	/		*	/	/	RNA polymerase
***orf219***	/	/	*	/	*	/	LAGLIDADG endonuclease
***orf250***	/	/	/	*	/	/	DNA polymerase
***orf283***	*	/	*	/	/	/	DNA polymerase
***orf294***	*	*	/	/	/	*	hypothetical protein
***orf309***	/	/	/	*	/	/	LAGLIDADG endonuclease
***orf396***	*	*	*	/	*	*	DNA polymerase
***orf413***	/	/		/	*	/	hypothetical protein
***orf429***	*(*orf439*)	*	*	/	*	*	hypothetical protein
***orf568***	*	*	*	/	*	*	RNA polymerase
***orf744***	/	/	/	/	*	/	DNA polymerase
***orf879***	/	/	/	/	*	/	DNA polymerase

The ‘*’ indicates that the non-conserved PCG or corresponding homologous gene exists in the *Ganoderma* species, and the ‘/’ indicates that there is no non-conserved PCG or homologous gene in this species. The *G. lucidum* (KC763799) and *G. lingzhi* s62 have orf439 and orf167 homologous to orf429 and orf151, respectively, according to their similarities in the amino acid sequence.

## Data Availability

The four *Ganoderma* mitogenomes, including *G. subamboinense* s118, *G. lucidum* s37, *G. lingzhi* s62, and *G. lingzhi* s74, were submitted to GenBank under the accession numbers MW752412, MW752414, MW752415, and MW752413, respectively. All data generated or analyzed during this study are included in this published article and its Supplementary Materials.

## Supplementary Materials

The following supporting information can be downloaded at: https://www.mdpi.com/article/10.3390/jof8080781/s1. Table S1: Comparison on mitogenomes among Ganoderma species; Table S2: Characterization of the mitogenome of four Ganoderma species; Table S3: Start and stop codon analyses of 15 core protein-coding genes in 13 Ganoderma species; Table S4: Codon usage analysis of the four Ganoderma mitogenomes; Table S5: Local BLAST analysis of the four Ganoderma mitogenomes against themselves; Table S6: Tandem repeats detected in the four Ganoderma mitogenomes using the online program Tandem Repeats Finder; Table S7: Species, and GenBank accession number used for phylogenetic analysis in this study.

## Abbreviations

Mitogenome (mitochondrial genome), PCG (protein-coding gene), Pcls (position classes), *Ks* (synonymous substitution rates), *Ka* (nonsynonymous substitution rates), BI (Bayesian inference), and ML (Maximum likelihood).

## References

[1] Kwon O.-C., Park Y.-J., Kim H.-I., Kong W.-S., Cho J.-H., Lee C.-S. (2016). Taxonomic Position and Species Identity of the Cultivated Yeongji ‘*Ganoderma lucidum*’ in Korea. Mycobiology.

[2] Jargalmaa S., Eimes J.A., Park M.S., Park J.Y., Oh S.-Y., Lim Y.W. (2017). Taxonomic evaluation of selected *Ganoderma* species and database sequence validation. PeerJ.

[3] Kües U., Nelson D.R., Liu C., Yu G.-J., Zhang J., Li J., Wang X.-C., Sun H. (2015). Genome analysis of medicinal *Ganoderma* spp. with plant-pathogenic and saprotrophic life-styles. Phytochemistry.

[4] Sun Y.-F., Xing J.-H., He X.-L., Wu D.-M., Song C.-G., Liu S., Vlasák J., Gates G., Gibertoni T., Cui B.-K. (2022). Species diversity, systematic revision and molecular phylogeny of *Ganodermataceae* (*Polyporales, Basidiomycota*) with an emphasis on Chinese collections. Stud. Mycol..

[5] Wang X.-C., Xi R.-J., Li Y., Wang N.-M., Yao Y.-J. (2012). The Species Identity of the Widely Cultivated *Ganoderma*, ‘*G. lucidum*’ (*Ling-zhi*), in China. PLoS ONE.

[6] Dhillon B., Hamelin R.C., Rollins J.A. (2021). Transcriptional profile of oil palm pathogen, *Ganoderma boninense*, reveals activation of lignin degradation machinery and possible evasion of host immune response. BMC Genom..

[7] Hidayati N., Glen M., Nurrohmah S.H., Rimbawanto A., Mohammed C.L. (2014). *Ganoderma steyaertanum* as a root-rot pathogen of forest trees. For. Pathol..

[8] Page D.E., Glen M., Puspitasari D., Rimbawanto A., Ratkowsky D., Mohammed C. (2017). Sexuality and mating types of *Ganoderma philippii*, *Ganoderma mastoporum* and *Ganoderma australe*, three basidiomycete fungi with contrasting ecological roles in south-east Asian pulpwood plantations. Australas. Plant Pathol..

[9] Francis A., Beadle C., Puspitasari D., Irianto R., Agustini L., Rimbawanto A., Gafur A., Hardiyanto E., Junarto N., Hidyati B. (2014). Disease progression in plantations of *Acacia mangium* affected by red root rot (*Ganoderma philippii*). For. Pathol..

[10] Bishop K.S., Kao C.H., Xu Y., Glucina M.P., Paterson R.R.M., Ferguson L.R. (2015). From 2000 years of *Ganoderma lucidum* to recent developments in nutraceuticals. Phytochemistry.

[11] Paterson R.R.M. (2006). Ganoderma—A therapeutic fungal biofactory. Phytochemistry.

[12] Zhu Y., Xu J., Sun C., Zhou S., Xu H., Nelson D.R., Qian J., Song J., Luo H., Xiang L. (2015). Chromosome-level genome map provides insights into diverse defense mechanisms in the medicinal fungus *Ganoderma sinense*. Sci. Rep..

[13] Chan J.S., Asatiani M.D., Sharvit L.E., Trabelcy B., Barseghyan G.S., Wasser S.P. (2015). Chemical Composition and Medicinal Value of the New *Ganoderma tsugae* var. jannieae CBS-120304 Medicinal Higher Basidiomycete Mushroom. Int. J. Med. Mushrooms.

[14] Chien R.C., Tsai S.Y., Lai E.Y., Mau J.L. (2015). Antiproliferative Activities of Hot Water Extracts from Culinary-Medicinal Mush-rooms, *Ganoderma tsugae* and *Agrocybe cylindracea* (Higher Basidiomycetes) on Cancer Cells. Int. J. Med. Mushrooms.

[15] Dai Y.-C., Zhou L.-W., Hattori T., Cao Y., Stalpers J.A., Ryvarden L., Buchanan P., Oberwinkler F., Hallenberg N., Liu P.-G. (2017). *Ganoderma Lingzhi* (*Polyporales*, *Basidiomycota*): The scientific binomial for the widely cultivated medicinal fungus *Lingzhi*. Mycol. Prog..

[16] Dai Y.C., Yang Z.L., Cui B.K., Wu G., Yuan H.S., Zhou L.W., He S.H., Ge Z.W., Wu F., Wei Y.L. (2021). Diversity and sys-tematics of the important macrofungi in Chinese forests. Mycosystema.

[17] Gao X., Qi J., Ho C.-T., Li B., Mu J., Zhang Y., Hu H., Mo W., Chen Z., Xie Y. (2020). Structural characterization and immunomodulatory activity of a water-soluble polysaccharide from *Ganoderma leucocontextum* fruiting bodies. Carbohydr. Polym..

[18] Zheng S., Zhu N., Shi C. (2020). Genomic data mining approaches for the discovery of anticancer peptides from *Ganoderma* sinense. Phytochemistry.

[19] Liao B., Chen X., Han J., Dan Y., Wang L., Jiao W., Song J., Chen S. (2015). Identification of commercial *Ganoderma* (*Lingzhi*) species by ITS2 sequences. Chin. Med..

[20] Wang D.-M., Wu S.-H., Yao Y.-J. (2014). Clarification of the Concept of *Ganoderma* orbiforme with High Morphological Plasticity. PLoS ONE.

[21] Cao Y., Wu S.-H., Dai Y.-C. (2012). Species clarification of the prize medicinal *Ganoderma* mushroom “*Lingzhi*”. Fungal Divers..

[22] Cameron S.L. (2014). Insect Mitochondrial Genomics: Implications for Evolution and Phylogeny. Annu. Rev. Èntomol..

[23] Yuan M.-L., Zhang Q.-L., Guo Z.-L., Wang J., Shen Y.-Y. (2015). Comparative mitogenomic analysis of the superfamily *Pentatomoidea* (*Insecta: Hemiptera: Heteroptera*) and phylogenetic implications. BMC Genomics.

[24] Li J., Zhang J., Chen H., Chen X., Lan J., Liu C. (2013). Complete Mitochondrial Genome of the Medicinal Mushroom *Ganoderma lucidum*. PLoS ONE.

[25] Li Q., Xiang D., Wan Y., Wu Q., Wu X., Ma C., Song Y., Zhao G., Huang W. (2019). The complete mitochondrial genomes of five important medicinal *Ganoderma* species: Features, evolution, and phylogeny. Int. J. Biol. Macromol..

[26] Wang X.-C., Shao J., Liu C. (2015). The complete mitochondrial genome of the medicinal fungus *Ganoderma* applanatum (*Polyporales*, *Basidiomycota*). Mitochondrial DNA Part A.

[27] Wang X.-C., Wu K., Chen H., Shao J., Zhang N., Chen X., Lan J., Liu C. (2015). The complete mitochondrial genome of the white-rot fungus *Ganoderma meredithiae* (*Polyporales*, *Basidiomycota*). Mitochondrial DNA Part A.

[28] Latorre-Pellicer A., Moreno-Loshuertos R., Lechuga-Vieco A.V., Sánchez-Cabo F., Torroja C., Acín-Pérez R., Calvo E., Aix E., González-Guerra A., Logan A. (2016). Mitochondrial and nuclear DNA matching shapes metabolism and healthy ageing. Nature.

[29] Boore J.L. (1999). Animal mitochondrial genomes. Nucleic Acids Res..

[30] Cieslak M., Pruvost M., Benecke N., Hofreiter M., Morales A., Reissmann M., Ludwig A. (2010). Origin and History of Mitochondrial DNA Lineages in Domestic Horses. PLoS ONE.

[31] Li Q., Ren Y., Shi X., Peng L., Zhao J., Song Y., Zhao G. (2019). Comparative Mitochondrial Genome Analysis of Two Ectomycorrhizal Fungi (Rhizopogon) Reveals Dynamic Changes of Intron and Phylogenetic Relationships of the Subphylum Agaricomycotina. Int. J. Mol. Sci..

[32] Ye J., Cheng J., Ren Y., Liao W., Li Q. (2020). The First Mitochondrial Genome for *Geastrales* (*Sphaerobolus stellatus*) Reveals Intron Dynamics and Large-Scale Gene Rearrangements of *Basidiomycota*. Front. Microbiol..

[33] Zhang J.-Y., Zhang L.-P., Yu D.-N., Storey K.B., Zheng R.-Q. (2018). Complete mitochondrial genomes of Nanorana taihangnica and *N. yunnanensis* (Anura: *Dicroglossidae*) with novel gene arrangements and phylogenetic relationship of Dicroglossidae. BMC Evol. Biol..

[34] Barr C.M., Neiman M., Taylor D.R. (2005). Inheritance and recombination of mitochondrial genomes in plants, fungi and animals. New Phytol..

[35] Freel K.C., Friedrich A., Schacherer J. (2015). Mitochondrial genome evolution in yeasts: An all-encompassing view. FEMS Yeast Res..

[36] Li Q., Wang Q., Jin X., Chen Z., Xiong C., Li P., Liu Q., Huang W. (2018). Characterization and comparative analysis of six complete mitochondrial genomes from ectomycorrhizal fungi of the *Lactarius* genus and phylogenetic analysis of the *Agaricomycetes*. Int. J. Biol. Macromol..

[37] Li Q., Wang Q., Chen C., Jin X., Chen Z., Xiong C., Li P., Zhao J., Huang W. (2018). Characterization and comparative mitogenomic analysis of six newly sequenced mitochondrial genomes from ectomycorrhizal fungi (*Russula*) and phylogenetic analysis of the *Agaricomycetes*. Int. J. Biol. Macromol..

[38] Li Q., Wu P., Li L., Feng H., Tu W., Bao Z., Xiong C., Gui M., Huang W. (2021). The first eleven mitochondrial genomes from the ectomycorrhizal fungal genus (*Boletus*) reveal intron loss and gene rearrangement. Int. J. Biol. Macromol..

[39] Zhang Y.-J., Yang X.-Q., Zhang S., Humber R.A., Xu J. (2017). Genomic analyses reveal low mitochondrial and high nuclear diversity in the cyclosporin-producing fungus *Tolypocladium inflatum*. Appl. Microbiol. Biotechnol..

[40] Zhang Y.-J., Zhang H.-Y., Liu X.-Z., Zhang S. (2017). Mitochondrial genome of the nematode endoparasitic fungus *Hirsutella vermicola* reveals a high level of synteny in the family *Ophiocordycipitaceae*. Appl. Microbiol. Biotechnol..

[41] Sandor S., Zhang Y., Xu J. (2018). Fungal mitochondrial genomes and genetic polymorphisms. Appl. Microbiol. Biotechnol..

[42] Wang Y., Xu J. (2020). Mitochondrial Genome Polymorphisms in the Human Pathogenic Fungus *Cryptococcus neoformans*. Front. Microbiol..

[43] Zhang S., Wang X.-N., Zhang X.-L., Liu X.-Z., Zhang Y.-J. (2017). Complete mitochondrial genome of the endophytic fungus *Pestalotiopsis fici*: Features and evolution. Appl. Microbiol. Biotechnol..

[44] Li Q., Yang L., Xiang D., Wan Y., Wu Q., Huang W., Zhao G. (2019). The complete mitochondrial genomes of two model ectomycorrhizal fungi (*Laccaria*): Features, intron dynamics and phylogenetic implications. Int. J. Biol. Macromol..

[45] Huang W., Feng H., Tu W., Xiong C., Jin X., Li P., Wang X., Li Q. (2021). Comparative Mitogenomic Analysis Reveals Dynamics of Intron Within and Between *Tricholoma* Species and Phylogeny of *Basidiomycota*. Front. Genet..

[46] Chen C., Khaleel S.S., Huang H., Wu C.H. (2014). Software for pre-processing Illumina next-generation sequencing short read sequences. Source Code Biol. Med..

[47] Schubert M., Lindgreen S., Orlando L. (2016). AdapterRemoval v2: Rapid adapter trimming, identification, and read merging. BMC Res. Notes.

[48] Dierckxsens N., Mardulyn P., Smits G. (2017). NOVOPlasty: De novo assembly of organelle genomes from whole genome data. Nucleic Acids Res..

[49] Valach M., Burger G., Gray M., Lang B.F. (2014). Widespread occurrence of organelle genome-encoded 5S rRNAs including permuted molecules. Nucleic Acids Res..

[50] Bernt M., Donath A., Jühling F., Externbrink F., Florentz C., Fritzsch G., Pütz J., Middendorf M., Stadler P.F. (2013). MITOS: Improved de novo metazoan mitochondrial genome annotation. Mol. Phylogenet. Evol..

[51] Li Q., Ren Y., Xiang D., Shi X., Zhao J., Peng L., Zhao G. (2020). Comparative mitogenome analysis of two ectomycorrhizal fungi (*Paxillus*) reveals gene rearrangement, intron dynamics, and phylogeny of basidiomycetes. IMA Fungus.

[52] Lohse M., Drechsel O., Bock R. (2007). OrganellarGenomeDRAW (OGDRAW): A tool for the easy generation of high-quality custom graphical maps of plastid and mitochondrial genomes. Curr. Genet..

[53] Wang X., Song A., Wang F., Chen M., Li X., Li Q., Liu N. (2020). The 206 kbp mitochondrial genome of *Phanerochaete carnosa* reveals dynamics of introns, accumulation of repeat sequences and plasmid-derived genes. Int. J. Biol. Macromol..

[54] Caspermeyer J. (2016). MEGA Evolutionary Software Re-Engineered to Handle Today’s Big Data Demands. Mol. Biol. Evol..

[55] Rozas J., Ferrer-Mata A., Sánchez-DelBarrio J.C., Guirao-Rico S., Librado P., Ramos-Onsins S.E., Sánchez-Gracia A. (2017). DnaSP 6: DNA Sequence Polymorphism Analysis of Large Data Sets. Mol. Biol. Evol..

[56] Stothard P. (2000). The sequence manipulation suite: JavaScript programs for analyzing and formatting protein and DNA sequences. Biotechniques.

[57] Benson G. (1999). Tandem repeats finder: A program to analyze DNA sequences. Nucleic Acids Res..

[58] Chen C., Li Q., Fu R., Wang J., Deng G., Chen X., Lu D. (2021). Comparative mitochondrial genome analysis reveals intron dynamics and gene rearrangements in two *Trametes* species. Sci. Rep..

[59] Martin F.N., Bensasson D., Tyler B.M., Boore J.L. (2007). Mitochondrial genome sequences and comparative genomics of *Phytophthora ramorum* and *P. sojae*. Curr. Genet..

[60] Cheng J., Luo Q., Ren Y., Luo Z., Liao W., Wang X., Li Q. (2021). Panorama of intron dynamics and gene rearrangements in the phylum *Basidiomycota* as revealed by the complete mitochondrial genome of *Turbinellus floccosus*. Appl. Microbiol. Biotechnol..

[61] Larkin M.A., Blackshields G., Brown N.P., Chenna R., McGettigan P.A., McWilliam H., Valentin F., Wallace I.M., Wilm A., Lopez R. (2007). Clustal W and Clustal X version 2.0. Bioinformatics.

[62] Férandon C., Moukha S., Callac P., Benedetto J.-P., Castroviejo M., Barroso G. (2010). The *Agaricus bisporus* cox1 Gene: The Longest Mitochondrial Gene and the Largest Reservoir of Mitochondrial Group I Introns. PLoS ONE.

[63] Li Q., Li L., Feng H., Tu W., Bao Z., Xiong C., Wang X., Qing Y., Huang W. (2021). Characterization of the Complete Mitochondrial Genome of Basidiomycete Yeast *Hannaella oryzae*: Intron Evolution, Gene Rearrangement, and Its Phylogeny. Front. Microbiol..

[64] Katoh K., Rozewicki J., Yamada K.D. (2017). MAFFT online service: Multiple sequence alignment, interactive sequence choice and visualization. Briefings Bioinform..

[65] Vaidya G., Lohman D.L., Meier R. (2011). SequenceMatrix: Concatenation software for the fast assembly of multi-gene datasets with character set and codon information. Cladistics.

[66] Lanfear R., Frandsen P.B., Wright A.M., Senfeld T., Calcott B. (2017). PartitionFinder 2: New Methods for Selecting Partitioned Models of Evolution for Molecular and Morphological Phylogenetic Analyses. Mol. Biol. Evol..

[67] Ronquist F., Teslenko M., van der Mark P., Ayres D.L., Darling A., Höhna S., Larget B., Liu L., Suchard M.A., Huelsenbeck J.P. (2012). MrBayes 3.2: Efficient Bayesian Phylogenetic Inference and Model Choice across a Large Model Space. Syst. Biol..

[68] Li Q., Yang M., Chen C., Xiong C., Jin X., Pu Z., Huang W. (2018). Characterization and phylogenetic analysis of the complete mitochondrial genome of the medicinal fungus *Laetiporus sulphureus*. Sci. Rep..

[69] Stamatakis A. (2014). RAxML version 8: A tool for phylogenetic analysis and post-analysis of large phylogenies. Bioinformatics.

[70] Costa G.G., Cabrera O.G., Tiburcio R.A., Medrano F., Carazzolle M.F., Thomazella D.P., Schuster S.C., Carlson J.E., Guiltinan M.J., Bailey B.A. (2012). The mitochondrial genome of *Moniliophthora roreri*, the frosty pod rot pathogen of cacao. Fungal Biol..

[71] Al-Reedy R.M., Malireddy R., Dillman C.B., Kennell J.C. (2012). Comparative analysis of *Fusarium* mitochondrial genomes reveals a highly variable region that encodes an exceptionally large open reading frame. Fungal Genet. Biol..

[72] Basse C.W. (2010). Mitochondrial inheritance in fungi. Curr. Opin. Microbiol..

[73] Deng Y., Hsiang T., Li S., Lin L., Wang Q., Chen Q., Xie B., Ming R. (2018). Comparison of the Mitochondrial Genome Sequences of Six *Annulohypoxylon stygium* Isolates Suggests Short Fragment Insertions as a Potential Factor Leading to Larger Genomic Size. Front. Microbiol..

[74] Wu P., Bao Z., Tu W., Li L., Xiong C., Jin X., Li P., Gui M., Huang W., Li Q. (2020). The mitogenomes of two saprophytic Boletales species (*Coniophora*) reveals intron dynamics and accumulation of plasmid-derived and non-conserved genes. Comput. Struct. Biotechnol. J..

[75] Adams K.L., Palmer J.D. (2003). Evolution of mitochondrial gene content: Gene loss and transfer to the nucleus. Mol. Phylogenet. Evol..

[76] Adams K.L., Qiu Y.-L., Stoutemyer M., Palmer J.D. (2002). Punctuated evolution of mitochondrial gene content: High and variable rates of mitochondrial gene loss and transfer to the nucleus during angiosperm evolution. Proc. Natl. Acad. Sci. USA.

[77] Wang X., Jia L., Wang M., Yang H., Chen M., Li X., Liu H., Li Q., Liu N. (2020). The complete mitochondrial genome of medicinal fungus *Taiwanofungus camphoratus* reveals gene rearrangements and intron dynamics of *Polyporales*. Sci. Rep..

[78] Wang X., Wang Y., Yao W., Shen J., Chen M., Gao M., Ren J., Li Q., Liu N. (2020). The 256 kb mitochondrial genome of *Clavaria fumosa* is the largest among phylum *Basidiomycota* and is rich in introns and intronic ORFs. IMA Fungus.

[79] Li Q., He X., Ren Y., Xiong C., Jin X., Peng L., Huang W. (2020). Comparative Mitogenome Analysis Reveals Mitochondrial Genome Differentiation in Ectomycorrhizal and Asymbiotic Amanita Species. Front. Microbiol..

[80] Turmel M., Côté V., Otis C., Mercier J.P., Gray M., Lonergan K.M., Lemieux C. (1995). Evolutionary transfer of ORF-containing group I introns between different subcellular compartments (chloroplast and mitochondrion). Mol. Biol. Evol..

[81] Li Q., Chen C., Xiong C., Jin X., Chen Z., Huang W. (2018). Comparative mitogenomics reveals large-scale gene rearrangements in the mitochondrial genome of two *Pleurotus* species. Appl. Microbiol. Biotechnol..

[82] Lavrov D.V., Boore J.L., Brown W.M. (2002). Complete mtDNA Sequences of Two Millipedes Suggest a New Model for Mitochondrial Gene Rearrangements: Duplication and Nonrandom Loss. Mol. Biol. Evol..

[83] Zhong L., Wang M., Li D., Tang S., Zhang T., Bian W., Chen X. (2018). Complete mitochondrial genome of *Odontobutis haifengensis* (*Perciformes, Odontobutiae*): A unique rearrangement of tRNAs and additional non-coding regions identified in the genus *Odontobutis*. Genomics.

[84] Aguileta G., de Vienne D.M., Ross O.N., Hood M.E., Giraud T., Petit E., Gabaldón T. (2014). High Variability of Mitochondrial Gene Order among Fungi. Genome Biol. Evol..

[85] Hseu R.S., Wang H.H., Wang H.F., Moncalvo J.M. (1996). Differentiation and grouping of isolates of the *Ganoderma lucidum* com-plex by random amplified polymorphic DNA-PCR compared with grouping on the basis of internal transcribed spacer se-quences. Appl. Environ. Microbiol..

[86] Kuok Q.-Y., Yeh C.-Y., Su B.-C., Hsu P.-L., Ni H., Liu M.-Y., Mo F.-E. (2013). The triterpenoids of *Ganoderma tsugae* prevent stress-induced myocardial injury in mice. Mol. Nutr. Food Res..

[87] Hsu W.-H., Qiu W.-L., Tsao S.-M., Tseng A.-J., Lu M.-K., Hua W.-J., Cheng H.-C., Hsu H.-Y., Lin T.-Y. (2020). Effects of WSG, a polysaccharide from *Ganoderma lucidum*, on suppressing cell growth and mobility of lung cancer. Int. J. Biol. Macromol..

[88] Tel-Çayan G., Muhammad A., Deveci E., Duru M.E., Öztürk M. (2020). Isolation, structural characterization, and biological activities of galactomannans from *Rhizopogon luteolus* and *Ganoderma adspersum* mushrooms. Int. J. Biol. Macromol..

[89] Jiang L., Zhao L., Cheng D., Zhu L., Zhang M., Ruan Q., Chen W. (2017). The complete mitochondrial genome sequence of the Sichuan Digging Frog, *Kaloula rugifera* (Anura: *Microhylidae*) and its phylogenetic implications. Gene.

[90] Li W., Wang Z., Che Y. (2017). The Complete Mitogenome of the Wood-Feeding Cockroach *Cryptocercus meridianus* (*Blattodea*: *Cryptocercidae*) and Its Phylogenetic Relationship among Cockroach Families. Int. J. Mol. Sci..

[91] Li Q., Bao Z., Tang K., Feng H., Tu W., Li L., Han Y., Cao M., Zhao C. (2022). First two mitochondrial genomes for the order *Filobasidiales* reveal novel gene rearrangements and intron dynamics of *Tremellomycetes*. IMA Fungus.

[92] Binder M., Justo A., Riley R., Salamov A., Lopez-Giraldez F., Sjökvist E., Copeland A., Foster B., Sun H., Larsson E. (2013). Phylogenetic and phylogenomic overview of the *Polyporales*. Mycologia.

[93] Justo A., Miettinen O., Floudas D., Ortiz-Santana B., Sjökvist E., Lindner D., Nakasone K., Niemelä T., Larsson K.-H., Ryvarden L. (2017). A revised family-level classification of the *Polyporales* (*Basidiomycota*). Fungal Biol..

[94] Li H., Wu S., Ma X., Chen W., Zhang J., Duan S., Gao Y., Kui L., Huang W., Wu P. (2018). The Genome Sequences of 90 Mushrooms. Sci. Rep..

